# Hospital Meetings

**Published:** 1896-05-30

**Authors:** 


### HOSPITAL MEETINGS.

### CHELSEA HOSPITAL FOR WOMEN.

The annual meeting of the governors of the Chelsea Hospital for Women was held at the hospital on the 20th inst., Lord Glenesk, the chairman of the council, presiding. The Chairman congratulated the medical staff on the fact that, in spite of the many most important operations undertaken during the year 1895, the death-rate at the hospital was only 2·2 per cent. That was unprecedently low, but was beaten by the figures for the first four and a-half months of the year 1896, during which period, although the hospital had been fuller and the cases more severe, the death-rate was only 1 4 per cent. Turning to finance, Lord Glenesk pointed out that the hospital at the end of 1895 had a balance in hand of £193. A special appeal to provide for the year 1896 was made at the meeting with the result that £1,343 was subscribed. At the conclusion of the annual meeting a special meeting was held for the purpose of considering the following resolutions:—

> A member of the honorary medical staff may be suspended from duty for not longer than one calendar month by resolution of the weekly board, and notice of a special council meeting shall be immediately given to consider the case.
>
> A member of the honorary medical staff, whether previously suspended or otherwise, may be removed by a majority of not less than three-fourths of the members present at a council meeting specially convened, by not less than seven days' notice, which notice shall state that the meeting is to consider a matter concerning a member of the staff. The member of the staff in question shall have an opportunity of appearing before the council to give explanations. A member so removed shall *ipso facto* cease to be a member of the honorary medical staff.

These resolutions were carried unanimously on the motion of Lord Glenesk, seconded by Dr. Duncan, the senior member of the medical staff, who explained that it was with full concurrence of the medical staff that the two resolutions had been drawn up.

### VICTORIA HOSPITAL FOR CHILDREN.

Mr. Alfred Farquhar (the treasurer), in the absence of Lord Cadogan, presided over the annual meeting which was held at the hospital on the 20th inst. In moving the adoption of the annual report, the Chairman informed the meeting that Her Majesty the Queen had forwarded a donation of £50 to help to free the hospital from debt; H.R.H. the Princess Louise had sent £25; and H.R.H. the Duke of York, as chairman at the festival dinner, had contributed £25. The number of in-patients admitted during 1895 had been 1,130, of whom 420 were children of two years old and under. This, he considered, fully demonstrated the urgent necessity which existed for the addition to the hospital of the St. Gabriel's Ward for Infants. In the out-patient department, 18,770 new cases were registered, representing 56,628 attendances. During the year the working classes of Chelsea, Battersea, &c., through their Demonstration Committee, had contributed £126, making a total of £2,527 thus contributed by them to the hospital during the last thirteen years.

### THE HOSPITAL FOR WOMEN.

The fifty-third annual meeting was held at the hospital, Soho-square, on the 21st inst, the Duke of Westminster in the chair. The meeting was well attended, principally by ladies. To meet the increasing demands for indoor treatment the committee for the past two years have been asking the governors and subscribers for funds to provide additional in-patient accommodation. There are buildings adjacent to and belonging to the hospital now let on short agreements, which can be utilised for this purpose so soon as the necessary funds are forthcoming. The Chairman, in moving the adoption of the annual report, pointed out that though this increase was most desirable, yet the committee were quite wise not to attempt to do anything until the income of the hospital was more certain. Sir Rutherford Alcock, the chairman of the managing committee, in seconding, stated that out of one hundred very serious operation cases admitted to the hospital, cases which twenty years ago no medical man would have attempted to treat at all, only ten had resulted fatally, while in three hundred others, called minor operations, no death at all had occurred. The report states that with the object of securing a minimum allowance of at least one thousand cubic feet space to each bed throughout the hospital, the total number of beds has been reduced from sixty-six to sixty.

### ROYAL ORTHOPÆDIC HOSPITAL.

The adjourned annual meeting of the above hospital was held on the 21st inst. to receive the audited accounts, which, owing to the death of the late secretary, were not presented at the meeting earlier in the year. The accounts showed that the income for 1895 amounted to £1,812, and the expenditure to £2,014, leaving a debit balance of £202.

### ROYAL HOSPITAL FOR DISEASES OF THE CHEST.

In order to raise £4,000 for debts owing to the bankers and tradespeople, and to make provision for carrying on the work of the charity for the remainder of the year, a festival dinner in aid of the above hospital was held at the Whitehall Rooms on the 26th inst. Lord Charles Bruce occupied the chair, and in proposing "Success to the Hospital, briefly traced the history of the hospital. Although in its earliest days its growth was slow, yet the record of its work showed that it had been deserving of the support it had received. Founded at a time when there existed in this country no institution exclusively devoted to pulmonary diseases, it for many years was the sole refuge in London for patients suffering from that form of complaint. Its earliest designation was "The Infirmary for Diseases of the Lungs," and it was as unassuming in its pretensions as in its proportions. Its regulations, the chairman said, appeared to have been very practical and well suited to the exigencies of the time. Especially so was the following: "Ladies and nobility, members of parliament, the president and vice-presidents, medical men, and governors passing through a turnpike gate to the place of election of the court of governors may vote by proxy." The hospital appears to have been somewhat migratory in its character, for having been successively located in Bishopsgate and Finsbury, it at last found its home in the City Road. In 1863 a new building, which still forms part of the front of the hospital, was erected, and in 1866 emergency wards were built for out-patients. 1873 was a notable year in the history of the charity, for the governors at that date found that if the hospital was to minister to the wants of the suffering poor of the neighbourhood in any way commensurate with the demands made on the resources of the institution, it would be absolutely necessary for them to enlarge the institution, and they accordingly appointed a building committee in order to carry out that object. The opening of the new out-patient wing in 1877 enabled the council to increase the number of beds from ten to twenty. In 1880 the entire freehold of the hospital was acquired; three years later a very substantial addition was made to the building, and finally in 1886 the hospital was completed by the opening of a new wing, thus providing in all for eighty patients. A collection amounting to £3,400 was announced by the secretary as the result of the dinner.

